# Adipose Tissue-Derived Mesenchymal Stem Cells

**DOI:** 10.3390/cells10123433

**Published:** 2021-12-06

**Authors:** Bruce A. Bunnell

**Affiliations:** Department of Microbiology, Immunology and Genetics, University of North Texas Health Science Center, Fort Worth, TX 76107, USA; bruce.bunnell@unthsc.edu; Tel.: +1-817-735-2064

**Keywords:** adipose stem cells, biology, differentiation, secretome, human, clinical trials

## Abstract

The long-held belief about adipose tissue was that it was relatively inert in terms of biological activity. It was believed that its primary role was energy storage; however, that was shattered with the discovery of adipokines. Scientists interested in regenerative medicine then reported that adipose tissue is rich in adult stromal/stem cells. Following these initial reports, adipose stem cells (ASCs) rapidly garnered interest for use as potential cellular therapies. The primary advantages of ASCs compared to other mesenchymal stem cells (MSCs) include the abundance of the tissue source for isolation, the ease of methodologies for tissue collection and cell isolation, and their therapeutic potential. Studies conducted both in vitro and in vivo have demonstrated that ASCs are multipotent, possessing the ability to differentiate into cells of mesodermal origins, including adipocytes, chondrocytes, osteoblast and others. Moreover, ASCs produce a broad array of cytokines, growth factors, nucleic acids (miRNAs), and other macromolecules into the surrounding milieu by secretion or in the context of microvesicles. The secretome of ASCs has been shown to alter tissue biology, stimulate tissue-resident stem cells, change immune cell activity, and mediate therapeutic outcomes. The quality of ASCs is subject to donor-to-donor variation driven by age, body mass index, disease status and possibly gender and ethnicity. This review discusses adipose stromal/stem cell action mechanisms and their potential utility as cellular therapeutics.

## Biology of Adipose Tissue

Adipose tissue is found throughout the adult human body in bone marrow, intra-articular, subcutaneous, and visceral depots, as well as ectopic sites such as intra-hepatic and intra-muscular. For decades, adipose tissue was viewed as a passive organ that served primarily as an energy reservoir [1,2]. The discovery of the first cytokine produced by adipose tissue with systemic actions, leptin, led to the re-classification of adipose tissue as an endocrine organ [3]. Numerous additional adipokines have been subsequently identified, including adiponectin, omentin, and resistin, all produced by adipocytes. The adipokines play central roles in the regulation of metabolism. Adipose tissue also releases pro-inflammatory mediators, including IL-6, TNF-a, IL-1b, IL-8, and MCP-1, which drive inflammation [4,5]. It is believed that these cytokines are released by the non-adipocyte cells found in the adipose depot. 

Three types of adipose tissue have been described in humans:

A. White adipose tissue is primarily localized in subcutaneous depots or intra-abdominal depots is composed mainly of monolocular adipocytes and mainly functions as an energy storage depot that also produces adipokines.
B. Brown adipose tissue has energy storage activity, but thermogenic activity is induced by shivering and non-shivering mechanisms. A hallmark of brown adipose tissue is the expression of the mitochondrial membrane protein Uncoupling Protein 1 (UCP1), which regulates thermogenesis. Adipocytes in brown adipose tissue are more closely related to skeletal muscle than white adipose tissue [6,7,8].
C. Beige (“brite” or “brown/white”) adipose tissue serves as an energy storage depot that has the potential to express UCP1 and have thermogenic activity. However, from a developmental perspective, it is most similar to white adipose tissue [9].

Adipose tissue is composed mainly of mature adipocytes, though all adipose depots have considerable cellular heterogeneity. The heterogeneous population of cells includes preadipocytes, pericytes, endothelial cells, smooth muscle cells, fibroblasts, hematopoietic progenitor cells, an array of mature immune cells (B and T-lymphocytes, macrophages, myeloid cells) and adipose tissue-derived mesenchymal stem cells (ASCs). These cells make up a communication network that regulates the activity and function of adipose tissue depots.

The most common source of adipose tissue processed for stem cell isolation is subcutaneous white adipose tissue isolated from the abdomen, thigh, or hips/buttocks. When adipose tissue is broken down either by physical methods or enzymatic digestion, typically collagenase, the resulting cell pellet is denoted as the stromal vascular fraction (SVF). SVF is composed of all cell types found in adipose tissue, except for the adipocytes, which are depleted during the processing. The ASCs are the relatively homogenous population of spindle-shaped, fibroblast-like cells that are expanded out after 7–14 days of culture after SVF is plated onto standard cell culture surfaces. The ASCs can be expanded to large numbers and cryopreserved for future use.

1. Cell-Surface Antigen Profile of ASCs

Flow cytometric analysis of the cell surface cluster of differentiation (CD) antigens of ASCs is typically part of their characterization. Many of the CD antigens analyzed are canonical markers for mesenchymal stem cells. However, no ASC-specific CD marker has been identified that alone identifies a cell as an ASC. In general, analysis of both positive and negative CD antigens ensures that the isolated cells are mesenchymal stem cells and other cell lineages are not contaminating the preparation. Human ASCs express the traditional mesenchymal stem cell surface markers, including the cell adhesion molecules CD29, CD44, CD146, and CD166; the receptor molecules CD90 and CD105; and the GPI anchored enzyme CD73. In addition, ASCs should be negative (<2%) for the hematopoietic cell surface antigens, including CD11b, CD13, CD14, CD19, and CD45. They are also negative for the endothelial markers CD31 and HLA-DR [10].

ASCs are often reported to express CD34 in low-passage cultures. However, the expression decreases with the serial passage of the cells [11,12]. Thus, the expression of CD34 may depend on the passage number and the culture conditions used. In addition, ASCs do not express the adhesion marker CD106 or the sialoglycoprotein podocalyxin (PODXL), which makes them unique from bone marrow-derived MSCs (BMSCs) [10].

Cryopreservation of viable ASCs for long-term storage does not result in substantive alterations to the cell surface marker profile when thawed compared to freshly cultured cells. Moreover, ASCs isolated from young or old donors do not significantly differ in the cell surface antigen profile. However, obesity results in decreased expression of stemness markers compared to those isolated from lean donors [13]. 

Similar to other types of MSCs, ASCs are thought to be relatively immune-privileged because they do not express HLA-DR (major histocompatibility complex class II, MHC II) and low levels of MHC class I, which provides them with a significantly reduced immunogenicity over other cell types including fibroblasts [14]. However, ASCs have also been reported to be deficient in the expression of costimulatory molecules for T and B cells, including CD80, CD86, CD40, and its ligand, CD40L. In addition, when ASCs are used in mixed-lymphocyte reactions (MLR) in vitro, ASCs do not induce allogeneic T cells’ responses and can suppress levels of activation [10,14]. However, despite the belief that ASCs may be immune-privileged, they do not persist for protracted periods in vitro. In addition, they have been shown to induce complement pathways in vivo.

2. Colony-Forming Unit Potential and Multilineage Differentiation In Vitro

One of the primary characterization assays for ASCs is the analysis of self-renewal potential. The colony-forming unit-fibroblast (CFU-F) assay is the standard for defining the number of progenitor cells. In this assay, ASCs are plated at a low density, cultured for two weeks and their clonal expansion is assessed by staining with crystal violet to identify and visualize colonies. The number of colonies more significant than 2 mm in diameter (generally 50 cells or more) is counted as a readout of self-renewal efficiency. The self-renewal potential of ASCs is impacted by donor characteristics, such as age and obesity status. 

The ability to differentiate into various lineage-specific terminally differentiated cells is a hallmark of all stem cells. Thus, all MSCs, including ASCs, can efficiently differentiate into cells of mesodermal origin, including adipocytes, osteoblasts, and chondrocytes in vitro. Therefore, the characterization of the efficiency of differentiation by ASCs into bone, fat, and cartilage is a central component for their characterization. There have also been reports of ASCs efficiently differentiating along non-mesodermal lineages into various cell types in vitro, such as cardiomyocytes, neurons, and hepatocytes. However, the efficient differentiation along non-mesodermal lineages in vivo has not been reported for ASC.

3. ASC Secretome

Like other MSCs, ASCs produce a vast array of soluble mediators and extravascular vesicles (microvesicles) that can alter the biology of cell and tissues and mediate therapeutic effects in vivo. ASCs can influence the biology of target cells through products of the secretion of free macromolecules or the production of extracellular vesicles.

A. Soluble Mediators

The soluble trophic macromolecules produced by ASCs are composed of a broad array of cytokines, chemokines (both pro-inflammatory and anti-inflammatory), adipokines, antioxidative molecules, pro-angiogenic factors, anti-apoptotic factors, growth factors (VEGF, HGF, FGF, IGF-1), brain-derived neurotrophic factor (BDNF), interleukins (IL-1Ra, IL-6, IL-7, IL-8, IL-11). The medium from ASC cultures, denoted as conditioned medium, which contains all of the macromolecules secreted from ASCs (differentiated or not to a specific cell lineage), is considered a cell-free therapy for disease. In addition, several studies have demonstrated that the ASC conditioned media effectively mediates therapeutic benefits.

B. Extravascular Vesicles from ASC

Exosomes are small (40–200 nm diameter) lipid bilayer-bound extracellular vesicles derived from the endocytic pathway in eukaryotic cells. Exosomes have been isolated from numerous mammalian cell types, such as immune cells, muscle cells, neurons, and tumor cells. They are released into body fluids, including blood, saliva, cerebrospinal fluid, lymph, and urine. Exosomes play a central role in intercellular communication. Exosomes contain and transfer functional biomolecules, including proteins (e.g., enzymes and cytokines), nucleic acids, mRNAs, microRNAs (miRNAs), tRNAs, other non-coding RNAs, and lipids. Exosomes delivered in vivo influence processes like cell proliferation, migration and apoptosis, regulation of immune cell activity, angiogenesis, metabolism of cells, nerve regeneration, and tumorigenesis. In general, exosomes derived from MSCs from distinct tissue sources possess similar biological properties.

4. Clinical Applications of ASCs and ASC-Derived Products

ASCs are under investigation as a therapy for a variety of human diseases across the globe. They may represent a viable therapy in patients with various diseases and disorders, such as stroke, myocardial ischemia, multiple sclerosis, osteoarthritis, pulmonary diseases, acute kidney injury, and chronic wounds. For clinical applications, two strategies are being explored, the infusion of either autologous or allogeneic ASC. Autologous transplantation of ASCs is considered safer; however, the use of allogeneic cells offers the potential for a universal donor approach in which multiple patients with readily available, fully characterized, and optimized ASC are used for treatments. The advantage of the allogenic universal donor approach eliminates patient-specific ASC variability, enhances efficacy, and ensures reproducible efficacy results for patients with long-term therapy. To date, the vast majority of clinical trials have focused on autologous transplantation strategies.

A large number of preclinical studies testing the therapeutic potential ASCs have been performed in vitro and in vivo in preclinical animal models. The bulk of the findings reported have demonstrated encouraging results, which has resulted in human clinical trials testing, SVF, ASC, or ASC-derived exosomes in an array of diseases and disorders. 

Information collected from clinicaltrials.gov (accessed on 28 November 2021) in November of 2021 indicates that there are 440 clinical trials assessing the ability of ASCs to treat various diseases and disorders, of which 216 trials were categorized as not yet recruiting, recruiting or active, or unknown status. Of these trials, 71 are investigating SVF as therapy when searched across the same categories. The preponderance of clinical trials using SVF, ASC, or ASC-derived products are in the early stages, with more than 90% being categorized as early phase/phase 1, phase 1/2, or phase 2, and less than 10% categorized as phase 3 or phase 4. The top five categories or medical conditions under investigation are digestive system diseases, musculoskeletal, connective tissue and skin, cardiovascular, and central nervous system based on the numbers of trials in each category.

A. Osteoarthritis

Osteoarthritis (OA) is a degenerative disease in which there is a breakdown of cartilage and bone in the joints. OA typically manifests in the knees, hands, hips, feet, and spine. The knee is the primary problematic site in patients. Typical disease symptoms include pain and joint stiffness and swelling, which reduce the range of motion. SVF and ASCs have been investigated as an OA therapy in several clinical trials [15,16,17,18,19,20,21]. While the outcomes from these trials have been mixed, autologous ASCs have been shown to be safe and offer some improvement in disease-associated pain. Many patients have experienced improvements in pain, function, mobility, and overall quality of life on various clinical questionnaires after SVF and ASC administration into the affected joints. The clinical improvements persisted in these studies for several months to more than two years in many patients. However, reports of fundamental improvements in disease pathophysiology have not been reported.

B. Cardiovascular Disease

The leading cause of death worldwide is cardiovascular diseases (CVDs). Ischemic heart disease, such as myocardial infarction (MI), is the most common CVD. Several human clinical trials have been performed based on encouraging data with ASCs in preclinical animal models of CVD. The trials have focused on myocardial ischemia, MI, and heart failure (congestive and chronic). ASCs are effective at reducing myocardial dysfunction after myocardial ischemia. In preclinical research, SVF and ASCs improve functional parameters as demonstrated by increasing left ventricular ejection fraction (LVEF), fractional shortening, wall thickness, contractility, and six-minute walk test distance while decreasing left ventricular end-diastolic diameter, left ventricular end-systolic diameter, and overall remodeling [22,23,24,25,26,27]. Based on this encouraging data, several clinical trials have been performed. One of the initial trials, the APOLLO trial, evaluated the safety and feasibility of intracoronary infusion of ASCs in patients with ST-elevation MI [28]. Reported outcomes at six months included revascularization, improved heart function, and reduction in scarring in the heart. The ATHENA trial delivered ASCs via intramyocardial route into patients with severe chronic MI [29]. The data indicate that patients had elevated cardiac function and improvements in occluded blood perfusion. The ADVANCE trial is a phase 2 trial designed to assess the ability of ASCs to reduce infarct size in patients with ST-elevation acute MI. However, outcomes have not yet been published.

C. Multiple Sclerosis

Multiple sclerosis (MS) is a prevalent, chronic neurodegenerative disease driven by an aberrant inflammatory response against myelin, an integral component of the central nervous system (CNS). The immune attack on myelin results in demyelinated lesions in both the white and gray matter found in the spinal cord and brain. Although most MS patients are diagnosed with the relapsing-remitting form, the majority of patients eventually develop a progressive form of the disease exhibiting severe debilities [30,31]. Clinical trials investigating the infusion of SVF, and ASCs in MS patients have been reported [32,33]. One trial delivered autologous ASCs intravenously into patients with secondary progressive MS. At the same time, the cells were found to be safe but no beneficial outcomes were observed in the small numbers of patients in the trial [32]. Trial results were reported in 2019 in which patients were given intracerebroventricular (ICV) brain injections of autologous SVF as a potential therapy for Alzheimer’s disease (AD), amyotrophic lateral sclerosis (ALS), progressive multiple sclerosis (MS-P), Parkinson’s disease, spinal cord injury, traumatic brain injury, and stroke. Results pertaining specifically to the MS patients indicated that all of them were stable or showed signs of improvement [33]. Moreover, the intraventricular delivery of SVF appeared to be safe.

D. IBD/Crohn’s

ASCs have demonstrated the capacity to improve clinical symptoms and histologic disease activity in multiple studies of patients with severe IBD. Multiple clinical studies specifically included the patients’ refractory to antibiotics, immunomodulators, and surgical repair [34,35]. In this patient population, injection of ASCs around fistula enhanced the rate of fistula closure as assessed clinically and by MRI. ASCs even reduced the median time to clinical remission in one placebo-controlled study [35,36,37,38,39]. In most of these studies, at least 50% of patients treated with ASCs had complete closure of the treated fistula at the final time point of 240 days. In one study, 80% and 75% of patients (*n* = 36) still had complete fistula closure at 1 and 2 years after ASC treatment, respectively. ASCs have demonstrated the capacity to reduce clinical symptoms and histologic disease activity in multiple studies of patients with severe IBD.

ASC-derived exosomes and extracellular vesicles have been explored as an alternative to infusion intact cells to treat diseases. They have shown tremendous therapeutic promise in several preclinical studies by promoting tissue regeneration, regulating immune activity, and influencing tissue homeostasis in preclinical models. For example, a study conducted by Kim et al. demonstrated that CM of ASCs induced wound healing through stimulating the proliferation and migration of human dermal fibroblast and increasing collagen synthesis in vitro and in vivo. Another study demonstrated that exosomes collected from ASCs are beneficial in ischemia/reperfusion injury in the heart and kidneys. In addition, exosomes derived from ASCs accelerate cutaneous wound healing by increasing the synthesis of collagen. Other studies have shown that exosomes improve atopic dermatitis by decreasing the levels of inflammatory cells in the skin. Currently, there are no clinical trials using exosomes from ASC. Ongoing exosome research focuses on expanding the therapeutic data and delineating the macromolecules contained in the exosomes that mediate the therapeutic outcomes in vivo.

5. Conclusions and Future Directions

Adipose tissue depots are complex physiologic environments central to cellular activities, including energy metabolism, endocrinology, and immunity. Adipose tissue is a heterogeneous population of cells. Research indicates that both the heterogeneous SVF and the more homogeneous culture-expanded ASCs have therapeutic potential in regenerative medicine and possibly applications in tissue engineering. While the biologic properties of ASCs are still being understood, the cells are under clinical investigation in human trials for an array of diseases. However, a deeper understanding of the therapeutic potential of SVF or ASC-derived subpopulations, the secretome produced by the cells and the immunomodulatory capabilities may permit the isolation of application-specific ASCs. However, investigators must address several considerations regarding ASCs, including developing standardized methods for cell isolation, culture conditions, proliferation, differentiation and characterization methodology, and ensure safety and efficacy. Donor-to-donor variation significantly impacts the biologic and therapeutic potential of both SVF and ASCs regarding their differentiation, growth, and therapeutic effectiveness. More research into the impact of donor characteristics such as age, BMI, health status, environmental factors, ethnicity, and gender will allow for a deeper understanding of the therapeutic potential of ASCs.

## References

[1] Cawthorn W.P., Scheller E.L., MacDougald O.A. (2012). Adipose Tissue Stem Cells Meet Preadipocyte Commitment: Going Back to the Future. J. Lipid Res..

[2] Trayhurn P., Wood I.S. (2005). Signalling Role of Adipose Tissue: Adipokines and Inflammation in Obesity. Biochem. Soc. Trans..

[3] Zhang Y., Proenca R., Maffei M., Barone M., Leopold L., Friedman J.M. (1994). Positional Cloning of the Mouse Obese Gene and its Human Homologue. Nature.

[4] Caër C., Christine Rouault C., Le Roy T., Christine Poitou C., Aron-Wisnewsky J., Adriana Torcivia A., Bichet J.-C., Clément K., Michèle Guerre-Millo M., André S. (2017). Immune Cell-Derived Cytokines Contribute to Obesity-Related Inflammation, Fibrogenesis and Metabolic Deregulation in Human Adipose Tissue. Sci. Rep..

[5] Coppack S.W. (2001). Pro-Inflammatory Cytokines and Adipose Tissue. Proc. Nutr. Soc..

[6] Seale P. (2010). Transcriptional Control of Brown Adipocyte Development and Thermogenesis. Int. J. Obes..

[7] Seale P., Bjork B., Yang W., Kajimura S., Chin S., Kuang S., Scime A., Devarakonda S., Chin S., Conroe H.M. (2008). PRDM16 Controls a Brown Fat/Skeletal Muscle Switch. Nature.

[8] Seale P., Conroe H.M., Estall J., Kajimura S., Frontini A., Ishibashi J., Cohen P., Cinti S., Spiegelman B.M. (2011). Prdm16 determines the thermogenic program of subcutaneous white adipose tissue in mice. J. Clin. Investig..

[9] Wu J., Bostrom P., Sparks L.M., Ye L., Choi J.H., Giang A.H., Khandekar M., Nuutila P., Schaart G., Huang K. (2012). Beige adipocytes are a distinct type of thermogenic fat cell in mouse and human. Cell.

[10] Bourin P., Bunnell B.A., Casteilla L., Dominici M., Katz A.J., March K.L., Redl H., Rubin J.P., Yoshimura K., Gimble J.M. (2013). Stromal cells from the adipose tissue-derived stromal vascular fraction and culture expanded adipose tissue-derived stromal/stem cells: A joint statement of the International Federation for Adipose Therapeutics and Science (IFATS) and the International Society for Cellular Therapy (ISCT). Cytotherapy.

[11] Suga H., Matsumoto D., Eto H., Inoue K., Aoi N., Kato H., Araki J., Yoshimura K. (2009). Functional implications of CD34 expression in human adipose-derived stem/progenitor cells. (ORIGINAL RESEARCH REPORT)(cluster of differentiation)(Report). Stem Cells Dev..

[12] Traktuev D.O., Merfeld-Clauss S., Li J., Kolonin M., Arap W., Pasqualini R., Johnstone B.H., March K.L. (2008). A population of multipotent CD34-positive adipose stromal cells share pericyte and mesenchymal surface markers, reside in a periendothelial location, and stabilize endothelial networks. Circ. Res..

[13] Durandt C., Dessels C., da Silva C., Murdoch C., Pepper M.S. (2019). The effect of early rounds of *ex vivo* expansion and cryopreservation on the adipogenic differentiation capacity of adipose-derived stromal/stem cells. Sci. Rep..

[14] McIntosh K., Zvonic S., Garrett S., Mitchell J.B., Floyd Z.E., Hammill L., Kloster A., Halvorsen Y.D., Ting J.P., Storms R.W. (2006). The immunogenicity of human adipose-derived cells: Temporal changes in vitro. Stem Cells.

[15] Bui K.H.-T., Duong T.D., Nguyen N.T., Thanh Duc Nguyen T.D., Le V.T., Mai V.T., Phan N.L.-C., Le D.M., Ngoc N.K., Van Pham P. (2014). Symptomatic knee osteoarthritis treatment using autologous adipose derived stem cells and platelet-rich plasma: A clinical study. Biomed. Res. Ther..

[16] Koh Y.G., Choi Y.J., Kwon S.K., Kim Y.S., Yeo J.E. (2015). Clinical results and second-look arthroscopic findings after treatment with adipose-derived stem cells for knee osteoarthritis. Knee Surg. Sports Traumatol. Arthrosc..

[17] Gibbs N., Diamond R., Sekyere E.O., Thomas W.D. (2015). Management of knee osteoarthritis by combined stromal vascular fraction cell therapy, platelet-rich plasma, and musculoskeletal exercises: A case series. J. Pain Res..

[18] Pers Y.-M., Rackwitz L., Ferreira R., Pullig O., Delfour C., Barry F., Sensebe L., Casteilla L., Fleury S., Bourin P. (2016). Adipose mesenchymal stromal cell-based therapy for severe osteoarthritis of the knee: A phase I dose escalation trial. Stem Cells Trans. Med..

[19] Koh Y.G., Kwon O.R., Kim Y.S., Choi Y.J., Tak D.H. (2016). Adipose-derived mesenchymal stem cells with microfracture versus microfracture alone: 2-year follow-up of a prospective randomized trial. Arthroscopy.

[20] Nguyen P.D., Tran T.D.-X., Nguyen H.T.-N., Vu H.T., Le P.T.-B., Phan N.L.-C., Vu N.B., Phan N.K., Pham P.V. (2017). Comparative clinical observation of arthroscopic microfracture in the presence and absence of a stromal vascular fraction injection for osteoarthritis. Stem Cells Transl. Med..

[21] Yokota N., Yamakawa M., Shirata T., Kimura T., Kaneshima H. (2017). Clinical results following intra-articular injection of adipose derived stromal vascular fraction cells in patients with osteoarthritis of the knee. Regen. Ther..

[22] Otto Beitnes J., Øie E., Shahdadfar A., Karlsen T., Müller R.M.B., Aakhus S., Reinholt F.P., Brinchmann J.E. (2012). Intramyocardial injections of human mesenchymal stem cells following acute myocardial infarction modulate scar formation and improve left ventricular function. Cell Trans..

[23] Kim S.W., Lee D.-W., Yu L.-H., Zhang H.-Z., Kim C.E., Kim J.-M., Park T.-H., Cha K.-S., Seo S.-Y., Roh M.-S. (2012). Mesenchymal stem cells overexpressing GCP-2 improve heart function through enhanced angiogenic properties in a myocardial infarction model. Cardiovasc. Res..

[24] Li T.S., Smith R., Zhang Y., Sun B., Matsushita N., Cheng K., Malliaras K., Smith R.R., Blusztajn A., Terrovitis J. (2012). Direct comparison of different stem cell types and subpopulations reveals superior paracrine potency and myocardial repair efficacy with cardiosphere derived cells. J. Am. Coll. Cardiovasc..

[25] Paul A., Srivastava S., Chen G., Shum-Tim D., Prakash S. (2013). Functional assessment of adipose stem cells for xenotransplantation using myocardial infarction immunocompetent models: Comparison with bone marrow stem cells. Cell Biochem. Biophys..

[26] Shudo Y., Miyagawa S., Ohkura H., Fukushima S., Saito A., Shiozaki M., Kawaguchi N., Matsuura N., Shimizu T., Okano T. (2014). Addition of mesenchymal stem cells enhances the therapeutic effects of skeletal myoblast cell sheet transplantation in a rat ischemic cardiomyopathy model. Tissue Eng. Part A.

[27] Rasmussen J.G., Frøbert O., Holst-Hansen C., Kastrup J., Baandrup U., Zachar V., Fink T., Simonsen U. (2014). Comparison of human adipose-derived stem cells and bone marrow-derived stem cells in a myocardial infarction model. Cell Trans..

[28] Houtgraaf J.H., den Dekker W.K., van Dalen B.M., Springeling T., de Jong R., van Geuns R.J., Geleijnse M.L., Fernandez-Aviles F., Zijlsta F., Serruys P.W. (2012). First experience in humans using adipose tissue-derived regenerative cells in the treatment of patients with ST-segment elevation myocardial infarction. J. Am. Coll. Cardiol..

[29] Henry T.D., Pepine C.J., Lambert C.R., Traverse J.H., Schatz R., Costa M., Povsic T.J., Anderson R.D., Willerson J.T., Kesten S. (2017). The Athena trials: Autologous adipose-derived regenerative cells for refractory chronic myocardial ischemia with left ventricular dysfunction. Catheter. Cardiovasc. Interv..

[30] Dulamea A. (2015). Mesenchymal stem cells in multiple sclerosis—Translation to clinical trials. J. Med. Life.

[31] Filippi M., Bar-Or A., Piehl F., Preziosa P., Solari A., Vukusic S., Rocca M.A. (2018). Multiple Sclerosis. Nat. Rev..

[32] Fernandez O., Izquierdo G., Fernandez V., Leyva L., Reyes V., Guerrero M., Leon A., Arnaiz C., Navarro G., Paramo M.D. (2018). Adipose-derived mesenchymal stem cells (AdMSC) for the treatment of secondary-progressive multiple sclerosis: A triple blinded, placebo controlled, randomized phase I/II safety and feasibility study. PLoS ONE.

[33] Duma C., Kopyov O., Kopyov A., Berman M., Lander E., Elam M., Arata M., Weiland D., Cannell R., Caraway C. (2019). Human intracerebroventricular (ICV) injection of autologous, non-engineered, adipose-derived stromal vascular fraction (ADSVF) for neurodegenerative disorders: Results of a 3-year phase 1 study of 113 injections in 31 patients. Mol. Biol. Rep..

[34] Bernardi L., dos Santos C.H.M., Pinheiro V.A.S., Oliveira R.J., Antoniolli-Silva A.C.M.B. (2019). Transplantation of adipose-derived mesenchymal stem cells in refractory Crohn’s disease: Systematic review. Arq. Bras. Cir. Dig..

[35] Garcia-Arranz M., Herreros M.D., Gonzalez-Gomez C., De La Quintana P., Guadalajara H., Georgiev-Hristov T., Trebol J., Garcia-Olmo D. (2016). Treatment of Crohn’s-related rectovaginal fistula with allogeneic expanded-adipose derived stem cells: A phase I-IIa clinical trial. Stem Cells Transl. Med..

[36] De la Portilla F., Alba F., García-Olmo D., Herrerías J.M., González F.X., Galindo A. (2013). Expanded allogeneic adipose-derived stem cells (EASCs) for the treatment of complex perianal fistula in Crohn’s disease: Results from a multicenter phase I/IIa clinical trial. Int. J. Colorectal Dis..

[37] Cho Y.B., Lee W.Y., Park K.J., Kim M., Yoo H.-W., Yu C.S. (2013). Autologous adipose tissue-derived stem cells for the treatment of Crohn’s fistula: A phase I clinical study. Cell Transpl..

[38] Cho Y.B., Park K.J., Yoon S.N., Song K.H., Kim D.S., Jung S.H., Kim M., Jeong H.Y., Yu C.S. (2015). Long-term results of adipose-derived stem cell therapy for the treatment of Crohn’s fistula. Stem Cells Transl. Med..

[39] Park K.J., Ryoo S.-B., Kim J.S., Kim T.I., Baik S.H., Kim H.J., Lee K.Y., Kim M., Kim W.H. (2016). Allogeneic adipose derived stem cells for the treatment of perianal fistula in Crohn’s disease: A pilot clinical trial. Colorectal Dis..

